# Complete coding sequence of Tembusu virus isolated from geese in Taiwan in 2020

**DOI:** 10.1128/MRA.00256-23

**Published:** 2023-09-15

**Authors:** Yen-Ping Chen, Chih-Wei Huang, Fan Lee

**Affiliations:** 1 Veterinary Research Institute, Ministry of Agriculture, New Taipei City, Taiwan; Katholieke Universiteit Leuven, Leuven, Belgium

**Keywords:** Tembusu virus, coding sequence, phylogenesis, Taiwan

## Abstract

We reported the complete coding sequence of a Tembusu virus (TMUV) isolated from sick geese in Taiwan in 2020. The nucleotide sequence of the 20120008 isolate was most closely related to the strain TP1906 isolated from mosquitoes in Taiwan and clustered within a subgroup of Cluster 4 of the Tembusu virus.

## ANNOUNCEMENT

Tembusu virus (TMUV) belongs to the genus *Flavivirus* in the family *Flaviviridae*. TMUV was first discovered from *Culex tritaeniorhynchus* in Malaysia in 1955 and was isolated from diseased chicken in Malaysia in 2000 (1, 2). In 2010, a novel disease characterized by a severe drop in egg production and neurological signs in ducks and geese emerged in China and then spread to duck farms in Malaysia and Thailand. The etiological agent of the disease was also identified as TMUV (3–7). Moreover, a variant TMUV was discovered in *Culex penicula* and diseased ducks in Taiwan in 2019 (8, 9). Here, we report the complete coding sequence of TMUV isolate 20120008 obtained from a sick goose in Taiwan in 2020.

The 20120008 isolate, recovered from a diseased goose suffering from neurological disorders, was isolated via inoculation of the brain homogenate into the allantoic cavity of two 10-day-old embryonated Muscovy duck eggs. Viral RNA was extracted using a MagNA Pure Compact Nucleic Acid Isolation Kit I (Roche Diagnostics, Mannheim, Germany). A paired-end sequencing library was constructed using Illumina Stranded Total RNA Prep (Illumina, San Diego, California, USA) following the manufacturer’s protocol. Sequences were generated using a 500-cycle (2 × 250 bp paired-end) MiSeq reagent kit version 2 (Illumina) with MiSeq sequencer. Low- quality bases (<Q30) were trimmed using BBDuk implemented in Geneious Prime version 2022 (https://www.geneious.com). *De novo* assembly was performed using Geneious assembler. The longest consensus sequence was assembled by 799,450 reads, 6,180 bp in length and 18,668.3X for average sequence depth, identiﬁed as TMUV using BLASTN search. The highest similar strain, TMUV TP1906 strain (GenBank accession number MN747003), was used as the reference sequence for mapping. The final assembled sequence was used as a reference for further checking via mapping. Selected TMUV strains with complete ORFs were aligned using MAFFT version 7 (10, 11). Maximum-likelihood phylogeny was reconstructed using the Tamura-Nei substitution model (12) in Molecular Evolutionary Genetics Analysis version X (13) with 1,000 replicates of a bootstrap test.

**Fig 1:**
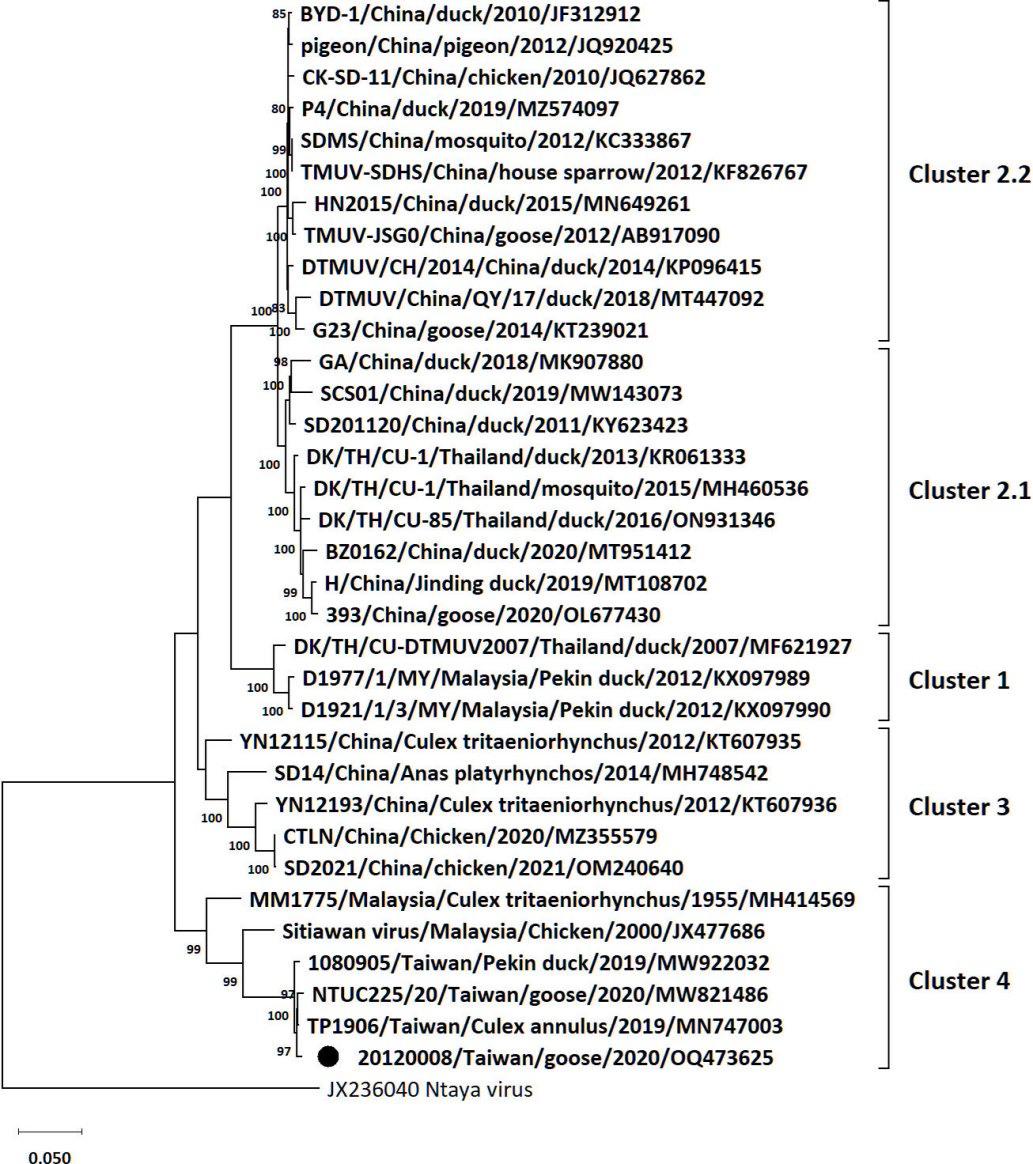
Maximum-likelihood phylogeny of Tembusu virus based on the complete polyprotein gene. Only bootstrap values (after 1,000 replicates) over 70% are indicated at each branch point as a percentage. For each virus strain, strain name, country, host, year of isolation or detection, and GenBank accession number are shown. The sequence of Tembusu virus 20120008 isolate is indicated with a black solid circle.

In total, 4,541,056 of 5,838,342 reads were assembled to the genome of the TMUV 20120008 isolate. The genome was 10,981 bp with 49% GC content and contains a single ORF encoding a polyprotein comprising 3,425 amino acids, a 85 bp 5’ untranslated region (UTR), and a 618 bp 3’ UTR. The average sequencing depth was 66,230.9X. Compared to the TMUV TP1906 strain, our sequence missed a 9 bp sequence in the 5’ UTR. The ORF sequence alignments revealed that the 20120008 isolate shared 86.91% to 99.53% nucleotide identities with the TMUV strains isolated in China, Thailand, Malaysia, and Taiwan, while it displayed the greatest similarity (99.53%) to the TMUV TP1906 strain isolated in Taiwan. The phylogeny showed that the 20120008 isolate was grouped into Cluster 4 (Fig. 1). Furthermore, the Taiwanese TMUV strains (20120008, TP1906, and 1080905), regardless of the isolated hosts, clustered together into the same subgroup of Cluster 4 (Fig. 1).

This reported sequence of the 20120008 isolate benefit further investigation on the epidemiology and evolution of the TMUV.

## Data Availability

Raw reads were deposited in SRA under accession number SRR23579292. The assembled genomic sequence of the TMUV 20120008 isolate has been deposited in GenBank under accession number OQ473625.
